# Comparison of Reverse Transcription Quantitative Real-Time PCR, Flow Cytometry, and Immunohistochemistry for Detection of Monoclonality in Lymphomas

**DOI:** 10.1155/2014/796210

**Published:** 2014-02-04

**Authors:** Anders Ståhlberg, Pierre Åman, Linda Strömbom, Neven Zoric, Alfredo Diez, Olle Nilsson, Mikael Kubista, Börje Ridell

**Affiliations:** ^1^Sahlgrenska Cancer Center, Sahlgrenska Academy, University of Gothenburg, 405 30 Gothenburg, Sweden; ^2^TATAA Biocenter, Odinsgatan 28, 411 03 Gothenburg, Sweden; ^3^Fujirebio Diagnostics AB, Elof Lindälvs Gata 13, 402 42 Gothenburg, Sweden; ^4^Institute of Biotechnology AS CR, Videnska 1083, 142 20 Prague 4, Czech Republic

## Abstract

In healthy humans, 60–70% of the B lymphocytes produce kappa light chains, while the remaining cells produce lambda light chains. Malignant transformation and clonal expansion of B lymphocytes lead to an altered kappa : lambda expression ratio, which is an important diagnostic criteria of lymphomas. Here, we compared three methods for clonality determination of suspected B cell lymphomas. Tumor biopsies from 55 patients with B cell malignancies, 5 B-lymphoid tumor cell lines, and 20 biopsies from patients with lymphadenitis were analyzed by immunohistochemistry, flow cytometry, and reverse transcription quantitative real-time PCR. Clonality was determined by immunohistochemistry in 52/53 cases, flow cytometry in 30/39 cases, and reverse transcription quantitative real-time PCR in 33/55 cases. In conclusion, immunohistochemistry was superior to flow cytometry and reverse transcription quantitative real-time PCR for clonality identification. Flow cytometry and reverse transcription quantitative real-time PCR analysis has complementary values. In a considerable number of cases tumor cells produced both kappa and lambda light chain transcripts, but only one type of light chain peptide was produced.

## 1. Introduction

B lymphocytes produce immunoglobulins consisting of a heavy chain and either a kappa (*IGKC*) or a lambda (*IGLC*) light chain. Each B lymphocyte decides early by the rearrangement of its immunoglobulin genes which light chain to produce [1]. The excluded light chain gene is not properly rearranged or remains in germ line configuration [2]. In healthy humans 60–70% of the B cells produce kappa chains and the rest produce lambda chains [1, 3]. Normal lymphoid tissues therefore contain a mixture of B cells that express *IGKC* and *IGLC* at a ratio of about 60 : 40 = 1.5.

Tumors of B cell origin are monoclonal and arise from one transformed cell. The single cell origin of malignant clones results in exclusive expression of *IGKC* or *IGLC* light chains in the vast majority of all B cell malignancies although B cell tumors that produce both kappa and lambda chains have been reported [4]. The clonal expression of *IGKC* or *IGLC* is thus used as an important diagnostic marker for B cell malignancies and currently determined on protein level by immunohistochemistry (IHC), flow cytometry (FC), or enzyme-linked immunosorbent assay techniques. Previously, we used reverse transcription quantitative real-time PCR (RT-qPCR) to quantify *IGKC* and *IGLC* gene transcripts in a small set of lymphomas and found that also gene expression level clonality was frequently evident [5]. In the present study we have used the same RT-qPCR method together with IHC and FC to analyze a larger cohort of 39 non-Hodgkin lymphomas, 16 chronic lymphatic leukemias, and 5 B cell derived tumor cell lines. The non-Hodgkin lymphomas consisted of 20 diffuse large B cell lymphomas, 16 follicular lymphomas, and 3 mantle cell lymphomas.

## 2. Material and Methods

### 2.1. Biopsies, Flow Cytometry, and Immunohistochemistry

The samples were transported from the operation theatre in ice-water-chilled boxes, handled in the laboratory within 30 min, and stored at −140°C. Parts of the tissues were fixed in formalin and embedded in paraffin according to the routine protocols of the pathology laboratory. Diagnosis was reached by a combination of microscopic histological evaluation, IHC of several markers, including the *κ* and *λ* chains, and in some cases by FC. Series of 5 *μ*m tissue sections were cut from each biopsy, deparaffinized, rehydrated, and stained with the following antibodies: rabbit anti-human kappa light chains and rabbit anti-human lambda chains (A0191 and A0194, DAKO). The antibodies were used at a dilution of 1 : 400 and bound antibodies were visualized using the second labeled antibody streptavidin biotin peroxidase system (DAKO). Stained sections were examined with a light microscope. For FC, a routine protocol for preparation of lymphoid cells from lymphoma tissue was used. Direct labeled antibodies specific for immunoglobulin kappa and lambda chains (A0191 and A0194, DAKO) were used for staining of recovered cells. First a gate for lymphocytes was set using forward and side scatter, followed by gates for kappa and lambda staining cells. We used a kappa : lambda ratio less than 0.4 or greater than 5 to prove FC monoclonality [6]. The samples were classified according to the Revised European-American Lymphoma and World Health Organization classification system.

### 2.2. RNA Extraction and RT-qPCR

RNA was extracted by use of the Fast Prep System (FastRNA Green; Qbiogene). We mixed 10 *μ*g of total RNA with 2 *μ*g of poly (dT) oligomers (Pharmacia) and incubated the mixture at 65°C for 5 min. First-strand cDNA synthesis was performed by adding 0.05 mol/L Tris-HCl (pH 8.3), 0.075 mol/L KCl, 3 mmol/L MgCl_2_, 0.01 mol/L dithiothreitol, 10 U/mL Moloney murine leukemia virus reverse transcriptase (Life Technologies), 0.05 units/mL RNA guard (Life Technologies), and 10 mmol/L of each deoxyribonucleotide (Life Technologies) to a final volume of 20 *μ*L and incubating the samples at 37°C for 1 h. The reaction was terminated by incubation at 65°C for 5 min, and samples were stored at −80°C. Real-time PCR measurements were performed on a Rotorgene 3000 (Corbett Research). Each PCR reaction contained 10 mmol/L Tris-HCl pH 8.3, 50 mmol/L KCl, 4 mM MgCl_2_, 400 *μ*mol/L each dNTP, 300 nmol/L each primer, 0.04 U/*μ*L Jumpstart Taq (Sigma), and 0.25x SYBR Green (Molecular Probes) in a 20 *μ*L reaction volume. The temperature profile was 95°C for 3 min followed by 40 cycles of amplification (95°C for 20 sec, 60°C for 20 sec, and 72°C for 20 sec). Primer sequences and detailed assay performance for *IGKC* and *IGLC* are reported elsewhere [5]. Formation of correctly sized PCR products was confirmed by agarose gel electrophoresis for all assays and melting curve analysis for all samples. RT-qPCR and statistical analysis of the data were performed as previously described [5]. A 95% confidence region for the *IGKC* : *IGLC *ratio using the negative lymphadenitis was used to prove RT-qPCR monoclonality.

### 2.3. RT-PCR Cloning and Sequencing

PCR products were obtained as for QPCR analysis and fractionated by agarose electrophoresis. Fragments were excised from the gels and used as templates for DNA sequencing as previously described [7].

## 3. Results and Discussion

The samples were analyzed by IHC, FC, and RT-qPCR for expression of IGKC and IGLC light chains (Tables 1 and 2). IHC indicated an exclusive or heavily dominant expression of IGKC or IGLC chains indicative of a monoclonal origin in all but one analyzed samples (98%). RT-qPCR analysis indicated a monoclonal dominance in 33 of 55 (60%) analyzed samples, while FC scored monoclonal dominance in 30 of 39 (77%) analyzed samples. Thus the IHC method was superior to FC and RT-qPCR in detecting monoclonality. The divergent results may in part be explained by the fact that biopsies, besides the tumor cells, often contain considerable numbers of normal lymphocytes that could contribute to the RNA samples analyzed by RT-qPCR and the cell population studied by flow cytometry. IHC analysis, on the other hand, allows for selective analysis of representative parts of the tumor tissue thus avoiding contribution of normal B lymphocytes. Furthermore, it is also known that 60–70% of non-Hodgkin lymphomas lack surface expression of IGLK and IGLC [8].

**Figure 1:**
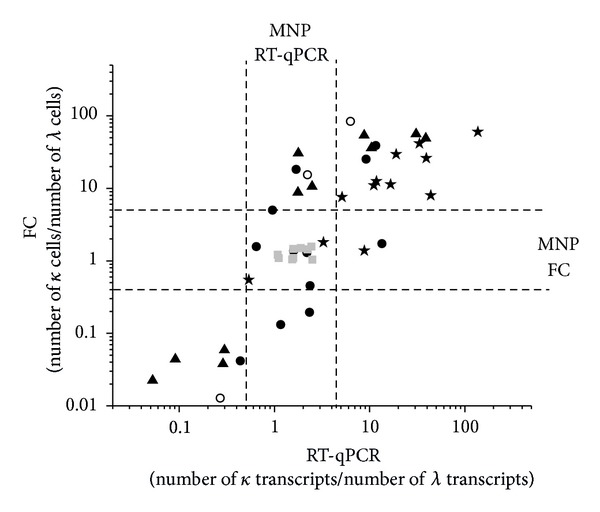
Comparison of IGKC : IGLC ratio between FC and RT-qPCR. The Pearson correlation coefficient is 0.65 (*P* < 0.01). The FC ratio refers to cell number, while the RT-qPCR ratio refers to transcript number. The areas where no monoclonality (MNP) could be proven are shown as dashed lines. Grey square, lymphadenitis; stars, diffuse large B cell lymphoma; triangles, chronic lymphocytic leukemia; dot, follicular lymphoma; circles, mantle cell lymphoma; *κ*, IGKC; *λ*, IGLC.

FC was more efficient than RT-qPCR in identifying clonal populations. However, there was an overall good correlation in expression levels of *IGKC* and *IGLC* between FC and RT-qPCR data despite the fact that FC data reflects cell number and RT-qPCR data reflects transcript numbers (Figure 1). In two cases (samples 135 and 168, Table 1), RT-qPCR detected clonal populations where FC failed. RT-qPCR may therefore serve as a valuable complement to IHC and FC in detection of monoclonal B cell populations. Moreover, RT-qPCR analysis can also be employed on minute fine needle aspirate samples that are not sufficient for flow cytometry or IHC and the same sample may also be analyzed simultaneously for expression of more marker genes.

**Figure 2:**
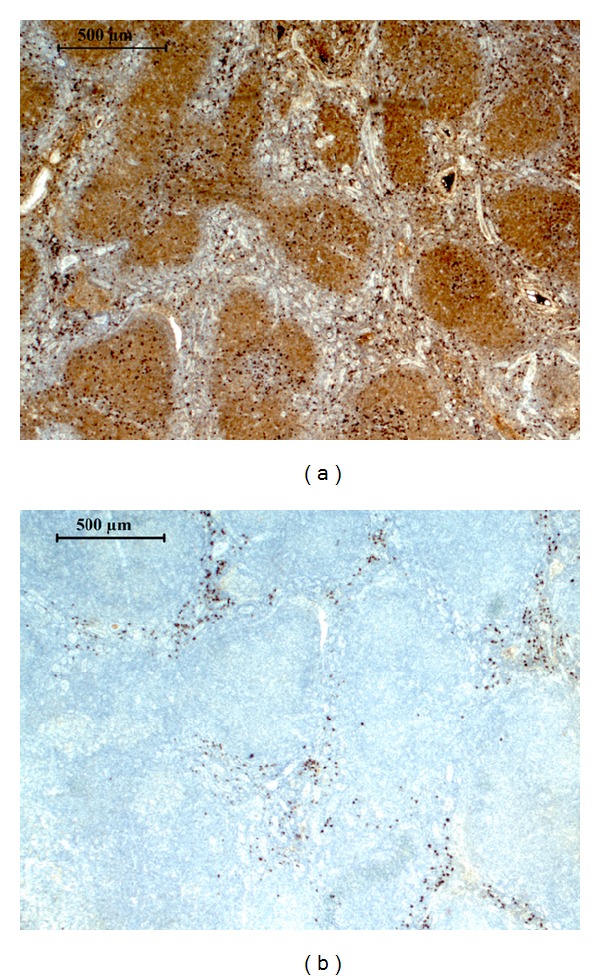
Immunohistochemistry of sample 165. The follicular lymphoma sample 165 was stained for (a) IGKC and (b) IGLC. Using RT-qPCR no clonality could be proven for this IGKC IHC positive sample.

Several of the *IGKC* and *IGLC* cotranscribing tumors appeared by IHC and FC to consist of homogenous tumor cell populations with no or few normal lymphocytes suggesting that some malignant B cell clones were dual producers of light chain mRNA (Figure 2). Dual production of *IGLC* chain mRNAs and proteins has been reported for various types of malignant B cell clones and normal B lymphocytes [4, 9]. These reports and our data prompted us to analyze five monoclonal B cell derived tumor cell lines (Table 3). These cell lines produce only one light chain species as analyzed by immunofluorescence, but four of them contained both *IGKC* and *IGLC* transcripts. To further confirm this result we isolated PCR products from two of the cell lines and sequence analysis showed that *IGKC* and *IGLC* transcripts were indeed produced simultaneously in these cell lines. Thus we conclude that human monoclonal B-lymphoid populations frequently simultaneously produce *IGKC* and *IGLC* transcripts.

We searched for possible correlations between dual light chain producers and tumor type. Dual producers were most common in the follicular lymphoma group in which 11/16 tumors failed to score for monoclonality based on RT-qPCR analysis. This could possibly be explained by larger abundance of normal cells in follicular lymphomas since FC showed highest failure rate for these samples, and may also depend on a higher frequency of dual producers.

Eleven of twelve cases where monoclonality was not detected by RT-qPCR produced IGKC protein as judged by IHC. This is more than expected from the normal ratio of IGKC and IGLC producers among normal B lymphocytes and lymphoma populations. In the present cohort of IHC investigated lymphomas, 30% were lambda producers. Thus, it is possible that dual mRNA producers arise more often among kappa protein producing cells. This is surprising since kappa chains are considered to be rearranged first and *IGLC* rearrangement is pursued only if *IGKC* rearrangement fails to produce a functional IGKC protein [10]. The activation of *IGLC* transcription is most likely a result of rearrangement that occurs after IGKC protein is produced and may therefore indicate a failure to inactivate the rearrangement mechanism. Failure to regulate a potentially risky DNA rearrangement process may also contribute to the frequent multiple genetic rearrangements in lymphomas. Age and gender were not correlated to the determined clonality (data not shown).

## 4. Conclusions

We conclude that IHC, FC, and RT-qPCR analysis of human B-lymphoid tumors gives partially divergent results. This may in part be explained by contribution of normal B lymphocytes into the tumor tissue. Our results also suggest that dual transcription of *IGKC* and *IGLC* loci is common in these tumors. This has to be taken in consideration when RT-qPCR analysis of *IGKC* and *IGLC* is used for diagnostic purpose. RT-qPCR remains an interesting complement for diagnostic purpose when only small samples, such as from fine needle cytology, are available.

## Figures and Tables

**Table 1:** Detailed patient information.

Sample ID	Classification	Gender	Age	FC	IHC	RT-qPCR
111	CLL	F	70	ND	Lambda	Lambda
112	CLL	F	82	ND	Lambda	MNP
113	CLL	M	58	Lambda	Lambda	Lambda
115	CLL	F	61	Lambda	Lambda	Lambda
116	CLL	F	57	Kappa	Kappa	Kappa
117	CLL	F	75	Kappa	Kappa	Kappa
118	CLL	F	85	Kappa	Kappa	Kappa
119	CLL	F	48	ND	Lambda	Lambda
131	CLL	M	72	ND	Lambda	Lambda
132	CLL	F	59	ND	Lambda	Lambda
145	CLL	F	76	Kappa	Kappa	MNP
146	CLL	M	46	Lambda	Lambda	Lambda
147	CLL	F	74	Kappa	Kappa	Kappa
149	CLL	F	76	Kappa	Kappa	MNP
189	CLL	F	65	Kappa	Kappa	MNP
191	CLL	M	60	Lambda	Lambda	Lambda
120	DLBL	M	80	ND	Kappa	MNP
121	DLBL	M	81	ND	Kappa	Kappa
123	DLBL	M	64	Kappa	Kappa	Kappa
124	DLBL	F	77	Kappa	Kappa	Kappa
125	DLBL	M	57	ND	MNP	Lambda
126	DLBL	M	70	Lambda	Lambda	Lambda
127	DLBL	M	70	Kappa	Kappa	Kappa
129	DLBL	M	58	ND	Kappa	MNP
133	DLBL	M	72	MNP	Lambda	MNP
135	DLBL	F	75	MNP	Kappa	Kappa
136	DLBL	F	78	ND	Kappa	MNP
137	DLBL	M	70	Kappa	Kappa	Kappa
138	DLBL	M	72	ND	Kappa	MNP
139	DLBL	M	33	Kappa	Kappa	Kappa
140	DLBL	F	78	Kappa	Kappa	kappa
153	DLBL	M	19	ND	Kappa	Kappa
156	DLBL	M	58	Kappa	Kappa	Kappa
182	DLBL	F	75	MNP	Kappa	MNP
184	DLBL	M	55	Kappa	Kappa	Kappa
186	DLBL	F	66	Kappa	Kappa	Kappa
157	FL	F	54	Kappa	Kappa	MNP
158	FL	F	51	ND	Kappa	MNP
159	FL	M	77	ND	Kappa	MNP
160	FL	F	60	ND	Lambda	MNP
161	FL	M	70	Lambda	Lambda	MNP
162	FL	M	69	Kappa	Kappa	Kappa
163	FL	M	52	Kappa	Kappa	Kappa
164	FL	M	53	ND	Kappa	Kappa
165	FL	F	59	Kappa	Kappa	MNP
166	FL	M	61	MNP	Kappa	MNP
167	FL	F	30	MNP	Lambda	MNP
168	FL	M	51	MNP	Kappa	Kappa
208	FL	F	83	MNP	ND	MNP
209	FL	F	55	Lambda	Lambda	Lambda
210	FL	F	46	MNP	Kappa	MNP
211	FL	M	64	Lambda	ND	MNP
204	MCL	M	61	Lambda	Lambda	Lambda
206	MCL	F	78	Kappa	Kappa	Kappa
207	MCL	F	70	Kappa	Kappa	MNP
101	LA	F	62	ND	MNP	Control
102	LA	M	25	ND	MNP	Control
103	LA	M	25	ND	MNP	Control
104	LA	M	16	ND	MNP	Control
105	LA	F	58	ND	MNP	Control
106	LA	M	59	ND	MNP	Control
107	LA	M	41	ND	MNP	Control
108	LA	M	7	ND	MNP	Control
109	LA	F	33	ND	MNP	Control
110	LA	F	28	ND	MNP	Control
192	LA	F	1	MNP	MNP	Control
193	LA	F	40	MNP	MNP	Control
194	LA	M	28	MNP	MNP	Control
195	LA	M	20	MNP	MNP	Control
196	LA	M	13	MNP	MNP	Control
197	LA	M	30	MNP	MNP	Control
198	LA	F	10	MNP	MNP	Control
199	LA	F	58	MNP	MNP	Control
200	LA	M	49	MNP	MNP	Control
201	LA	F	57	MNP	MNP	Control

CLL: chronic lymphocytic leukemia; DLBL: diffuse large B cell lymphoma; FL: follicular lymphoma; LA: lymphadenitis; F: female; M: male; MNP: monoclonality not proven; FC: flow cytometry; IHC: immunohistochemistry.

**Table 2:** Monoclonal populations detected in lymphoma and lymphadenitis biopsies^a^.

	RT-qPCR	FC	IHC
CLL	12/16	11/11	16/16
DLBL	14/20	10/13	19/20
FL	5/16	6/12	14/14
MCL	2/3	3/3	3/3
CLL/lymphoma total	33/55	30/39	52/53
LA	0/20	0/10	0/20

^a^Number of samples with clonality proven/total number of samples is shown. CLL: chronic lymphocytic leukemia; DLBL: diffuse large B cell lymphoma; FL: follicular lymphoma; MCL: mantle cell lymphoma; LA: lymphadenitis (polyclonal control).

**Table 3:** RT-qPCR data for 5 B-lymphoid tumor cell lines.

Cellinje	Kappa Cq	Lambda Cq	Kappa noRT Cq	Lambda noRT Cq	mRNA expression	IHC
U698	10.3	13.8	27	23.5	Kappa > Lambda	Kappa
DG75	10.4	15.9	25.2	24.2	Kappa > Lambda	Kappa
MC116	34.8	10.4	Not detected	23.5	Lambda only	Lambda
RPMI8226	12.2	14.4	25.1	22.2	Kappa > Lambda	Lambda
CCRF-SB	12.3	11.2	26.7	24.4	Lambda </*≈* Kappa	Kappa
Neg. control	>33	~30				

Cq: cycle of quantification (log transcript numbers); noRT: samples without reverse transcriptase; IHC: immunohistochemistry.
